# Prevalence of cardiovascular risk factors in an urban area of Togo: a WHO STEPS-wise approach in Lome, Togo

**DOI:** 10.5830/CVJA-2011-071

**Published:** 2012-07

**Authors:** S Baragou, M Djibril, B Atta, F Damorou, M Pio, A Balogou

**Affiliations:** Department of Cardiology, University of Lome, Lome, Togo; Department of Cardiology, University of Lome, Lome, Togo; Department of Cardiology, University of Lome, Lome, Togo; Department of Cardiology, University of Lome, Lome, Togo; Department of Cardiology, University of Lome, Lome, Togo; Department of Cardiology, University of Lome, Lome, Togo

**Keywords:** hypertension, risk factors, epidemiology, Togo, sub-Saharan Africa

## Abstract

**Objective:**

To determine the prevalence of hypertension and other cardiovascular risk factors in the general adult population of Lome.

**Methods:**

A cross-sectional household survey was conducted in Lome from October 2009 to January 2010, which focused on hypertension and other cardiovascular risk factors in 2 000 subjects 18 years and older. The World Health Organisation’s STEPS-wise approach on non-communicable diseases was used. During the first session, blood pressure (BP) was measured on three successive occasions, one minute apart, and the mean was recorded. A second measurement session was done three weeks later in patients with BP ≥ 140/90 mmHg during the first session. Hypertension was defined as BP > 140/90 mmHg after the second session, or on antihypertensive treatment. The other risk factors were studied by clinical and blood analysis.

**Results:**

We found 532 hypertensive patients out of a total of 2 000 subjects. The prevalence of hypertension was 26.6%. The mean age of hypertensive patients was 45 ± 10 years, ranging from 18 to 98 years. The prevalence of other cardiovascular risk factors was: stress (43%), sedentary lifestyle (41%), hypercholesterolaemia (26%), obesity (25.2%), hypertriglyceridaemia (21%), smoking (9.3%), alcohol use (11%) and diabetes (7.3%).

**Conclusions:**

The prevalence of hypertension and other cardiovascular risk factors in the population of Lome is high. These findings should draw the attention of authorities to define a national policy to combat hypertension and other cardiovascular risk factors.

## Abstract

The whole of sub-Saharan Africa and Togo in particular, have for some years seen an epidemiological transition, obligating people to face infectious transmissible diseases as well as non-transmissible ones, and particularly cardiovascular diseases. This epidemiological transition is largely associated with urbanisation and the change in lifestyle.^1^-^5^

In Togo, the prevalence of hypertension in hospital cardiology departments is 52%,^6^ but it is unknown in the general population. Cardiovascular diseases are the second cause of death in Togo after malaria.^6^ Knowledge of epidemiological data on cardiovascular risk factors is fundamental in order to define a national policy and combat cardiovascular diseases. The purpose of this study was therefore to determine the prevalence of hypertension and other cardiovascular risk factors in the general population of the urban area of Togo.

## Methods

A cross-sectional, prospective, community-based study was conducted in the town of Lome, which is subdivided into five districts and has approximately 1 056 200 inhabitants.^7^ Lome, the capital of Togo, is cosmopolitan and largely commercial, with an inter-tropical climate and varied socio-economic layers. It is located in southern Togo on the coast of the Atlantic Ocean.

The population of subjects aged 18 years and over was estimated at 497 098 at the time of the study.^7^ This work was conducted from 1 October 2009 to 31 January 2010 using the STEPS-wise WHO method on the monitoring of risk factors of non-communicable diseases.^8^ The General Director of Health and local administrative authorities in the town of Lome were informed of the period and the course of the investigation, and had issued their written authorisation. Fifteen investigators (10 medical assistants and five nurses), divided into five groups, were selected and trained. Supervision was provided by three physicians, including two cardiologists.

Calculation of sample size was done using the formula: *n* = Σ² × *P* × *Q/I* ² where Σ = standard deviation = 4, P = estimated prevalence of hypertension in Togo (20%), *Q* = 1–*P* = 0,8; *I* = precision index = 0.05. The final sample size was 2 000 subjects.

The sampling technique was random; 30 clusters were selected and 1 000 households were surveyed (34 households per cluster). In each household, one individual was recruited by gender, for a total of 68 individuals per cluster. Only subjects aged 18 years and over were examined. The investigators were careful to explain the purpose, importance and conduct of the study to participants. Verbal consent from each selected individual was acquired.

A preliminary investigation was done using the WHO questionnaire, adapted to the local situation.^9^ Each subject was first rested for 10 minutes and then questioned on the presence of diabetes, smoking and use of alcohol, sedentary or active lifestyle, stress and eating habits. Participants were also asked about the use of antihypertensive medication and those who were already on antihypertensive therapy were required to produce the drugs used.

Blood pressure (BP), weight and height were then measured, and the body mass index was calculated (kg/m^2^). BP was measured in both arms with a validated electronic BP monitor (Omron Inc), recommended for STEPS-wise surveys by the WHO.^8^ Three successive measurements were taken, one minute apart, and the mean was calculated. Any missing subjects were seen during a second visit.

A second measurement session was performed three weeks later in patients with BP ≥ 140/90 mmHg during the first session. Hypertension was defined as BP > 140/90 mmHg after the second measurement session,^10^ or on antihypertensive treatment, no matter what the blood pressure values were.

The other cardiovascular risk factors studied were obesity, alcohol use, smoking, stress, sedentary lifestyle and biological factors (diabetes, hypercholesterolaemia, hypertriglyceridaemia). Obesity was defined by a body mass index ≥ 30 kg/m^2^. Smoking was defined by a consumption of at least one cigarette daily, alcohol use by regular consumption of alcohol no matter the quantity, sedentary by lack of physical activity or less than 30 minutes of sport activity at least three times a week. Stress was evaluated with the rapid evaluation of Cunci^11^ and was defined as a score ≥ 30.

Blood analysis was done to determine glycaemia, total cholesterol and triglyceride levels. Some subjects refused blood sampling, and laboratory tests were therefore conducted only on those who accepted sampling, and no other selection criteria were used. In total, blood analyses were done for 1 012 (520 men and 492 women) of the 2 000 individuals involved in the study. The blood samples were analysed at the University Hospital of Lome, and a maximum of two hours between sampling and arrival of the samples at the laboratory was allowed.

Diabetes was defined as fasting glycaemia > 7 mmol/l (1.26 g/l) after two measures two weeks apart or > 11 mmol/l (2 g/l) during the first measure. Total hypercholesterolaemia was defined as cholesterolaemia > 5.16 mmol/l (2 g/l), and hypertriglyceridaemia as > 1.71 mmol/l (1.5 g/l).

## Statistical analysis

Confidence intervals (95% CI) and the chi-square (χ^2^) test were used to compare the prevalence of hypertension and other risk factors, with a significance set at *p* < 0.05. Epi Info 6.04 was used to record and analyse the data. Quantitative variables were reported as mean ± standard deviation and qualitative variables as percentages.

## Results

The total number of subjects included in the study was 2 000 (898 men and 1 102 women), with an overall mean age of 39 ± 10 years (40 ± 12 years for men, 38 ± 11 years for women), ranging from 18 to 98 years (Table 1). Forty-eight per cent of subjects had no school education, 28.5% had a primary level of education, 18.2% had secondary-level education, and 5.3% were university educated. According to diet, all respondents admitted to consuming fatty and processed foods, but not much vegetables and fruits.

**Table 1.** Characteristics Of The Study Population

*Age (years)*	*Male, n (%)*	*Female, n (%)*	*Overall, n (%)*
18–30	350 (38.9)	461 (42)	811 (40.5)
31–45	212 (23.6)	268 (24.3)	480 (24.1)
46–60	154 (17.1)	183 (16.6)	337 (16.8)
61–75	133 (14.8)	157 (14.2)	290 (14.5)
76–90	32 (3.5)	24 (2.2)	56 (2.8)
91–105	17 (1.9)	9 (0.8)	26 (1.3)
Overall	898 (100)	1102 (100)	2000 (100)

We identified 532 hypertensive individuals (243 men and 289 women). The prevalence of hypertension was 26.6% (25.7% in men and 27.6% in women, *p* = 0.09). Of the 532 hypertensive patients, 174 subjects (32.7%) were on antihypertensive treatment. Sixty-one per cent of hypertensive patients were classified as stage I, and 39% were classified as stage II, according to the JNCVII classification.^10^

The prevalence of hypertension increased with age (*p* < 0.001) (Table 2). The overall mean age of hypertensive individuals was 45 ± 10.4 years, with 45 ± 10.4 years for men and 46 ± 11.4 years for women. The mean systolic (SBP) and diastolic (DBP) blood pressure was 129.6 and 84.3 mmHg, respectively (Table 3).

**Table 2.** Prevalence Of Hypertension (%) By Gender And Age

	*Age groups (years)*					
*Gender*	*18–30*	*31–45*	*46–60*	*61–75*	*76–90*	*91–105*
Female	8	10.7	23	52.5	88.6	100
Male	9	17.3	28.4	51	75	88

**Table 3.** Mean Arterial Blood Pressure By Age Group

*Age (years)*	*Number*	*SBP (mmHg)*	*DBP (mmHg)*
18–30	811	121.7 ± 3.1	78.6 ± 2.4
31–45	337	127.7 ± 3.4	84.2 ± 2.5
46–60	480	136.1 ± 3.7	90.1 ± 3.4
61–75	290	147.2 ± 4.6	92.3 ± 3.8
76–90	56	150 ± 4.4	94 ± 3.4
91–105	26	152 ± 5.7	100 ± 4.8
Overall	2000	129.6 ± 3.5	84.3 ± 3.4

SBP = systolic blood pressure; DBP = diastolic blood pressure; mean ± standard deviation

Pre-hypertension, as defined by the JNCVII,^10^ was detected in 641 subjects (32%). The prevalence of other cardiovascular risk factors were stress (43%), sedentary lifestyle (41%), hypercholesterolaemia (26%), obesity (25.2%), hypertriglyceridaemia (21%), active smoking (9.3%), alcohol use (11%) and diabetes (7.3%) (Table 4)(Table 4).

**Table 4.** Other Cardiovascular Risk Factors, According To Gender

	*Male n (%)*	*Female n (%)*	*Overall n (%)*
Obesity*	150 (16.7)	354 (32.2)	504 (25.2)
Smoking	182 (20.2)	40 (3)	186 (9.3)
Sedentary lifestyle	192 (21.4)	628 (57)	820 (41)
Stress	420 (46.7)	440 (39.9)	860 (43)
Hypercholesterolaemia	114 (22)	149 (30.3)	263 (26)
Hypertriglyceridaemia	114 (21.9)	98 (19.9)	212 (21)
Alcohol use	165 (18.3)	55 (5)	220 (11)
Diabetes	36 (6.9)	38 (7.7)	74 (7.3)

*Android obesity: 18%; *gynoid obesity: 7.2% Sixty per cent of subjects had two or more risk factors.

Regarding professional groups, hypertension was observed among the unemployed (43%), civil servants (42.4%), housewives (54.2%), private-sector workers (23.8%), pensioners (56.2%) and farmers (6.3%). Forty-three per cent of hypertensive subjects had no education, 27.5% had a primary level of education, 18% had a secondary level of education, and 7.2% were university educated.

## Discussion

This research was done using the STEPS-wise approach of the WHO with regard to risk factors for non-communicable diseases at the population level.^8^ The sample was representative. This standard method of the WHO served as the official barometer in our particular context by which we collected comparable data and conducted reproducible surveys.

The main objective was to assess the current prevalence of hypertension and other cardiovascular risk factors in Lome, in order to assess the impact of individual preventive strategies in certain populations. The prospect of an investigation on a larger scale could determine the national prevalence of hypertension.

The average level of SBP of our respondents, 128.6 mmHg, was comparable to that reported in Morocco,^12^ at 129.8 mmHg. However, it was below the upper limit of 145 mmHg observed in Ouagadougou.^5^ The mean DBP was 84.6 mmHg in our study. The authors cited above^5^,^12^ found the respective values of 76 and 78 ± 12 mmHg in their studies.

SBP and the prevalence of hypertension increased with age, peaking at around 50 years.^1^-^5^ This was in agreement with what several other authors observed^1^-^5^,^9^ on the prevalence of hypertension in male participants. It should be noted that a slight female predominance of hypertension was inconsistently found in some African series: 21% of women versus 18.7% of men in rural Senegal,^13^ and 33% of women and 31% of men in Egypt.^14^

The overall prevalence of hypertension was 26.6% in Lome, explained partly by the new definition of hypertension used in this study, the urban area of residence of our respondents and their lifestyle, including their eating habits. Our result was lower than the overall prevalence of 32.9 and 33.6%, respectively, observed in urban areas at Ashanti in Ghana^1^ and in Morocco.^12^ However, the Eritrean investigation^15^ reported a prevalence of hypertension of 16.5%, reflecting the profile of these populations. These authors all used the current definition of hypertension.

Professional concerns, such as conditions of work and family life could explain the high prevalence of hypertension that we found among the unemployed, civil servants and housewives. In Zaire, Malu *et al*.^16^ reported a prevalence of hypertension of 35.5% among employees and 45.4% among housewives. At the autonomous port of Abidjan, 54.5% of hypertensive workers said they were stressed while they executed their duties.^17^

Educational level, according to Bertrand *et al*.^18^ was inversely associated with the occurrence of hypertension. In our respondents, 43% with no education were hypertensive, and 27.5% had a primary-level education. Literacy levels of hypertensive individuals, especially in Africa, represents a real challenge. It is imperative to lay particular emphasis on education and the change of lifestyle in pre-hypertensive subjects. It is also important to inform new patients with hypertension, even those who are asymptomatic, of the need for lifelong treatment.

Other cardiovascular risk factors were also present, namely stress, a sedentary lifestyle, dyslipidaemia, obesity and active smoking. These risk factors are modifiable, and a change of lifestyle is imperative. People need to change their eating habits by avoiding fatty foods and eating more vegetables and fruit.

These other risk factors, once detected, help prevent the occurrence of complications related to hypertension, and atherosclerosis. This could prevent the development from pre-hypertensive to hypertensive status. It should therefore be emphasised that the most important part of prevention, with little cost for state and communities, is based on the primary level. This involves educating people about the practices of a healthy lifestyle.

## Conclusion

The prevalence of hypertension and other cardiovascular risk factors in the general population is currently high in Lome. The situation is probably similar in other urban and suburban areas of Togo. These findings should draw the attention of the authorities to define a national policy to combat the development of hypertension and other cardiovascular risk factors. It is imperative to establish a national surveillance system for hypertension and other risk factors for atherosclerosis.

## References

[1] Cappucio FP, Micah FB, Emmert L (2004). Prevalence, detection, management and control of hypertension Ashanti, West Africa.. Hypertension.

[2] Van der Sande MA (2003). Cardiovascular disease in sub-Saharan Africa: a disaster waiting to happen.. Neth J Med.

[3] Addo J, Amoah AG, Koram KA (2006). The changing patterns of hypertension in Ghana: a study of four rural communities in the Ga District.. Dis Ethnol.

[4] Addo J, Smeeth L, Leon DA (2007). Hypertension in sub-Saharan Africa: a systematic review.. Hypertension.

[5] Niakara A, Fournet F, Gary J, Harang M, Nebie L, Salem G (2007). Hypertension, urbanization, social and spatial disparities: a crosssectional population-based survey in West African urban environment (Ouagadougou, Burkina Faso) Transactions of the Royal Society of Tropical. Med Hygiene.

[6] Baragou S, Yogui MY, Soussou B (2005). Aspects épidémiologiques des pathologies cardiovasculaires en milieu hospitalier à Lomé.. J Rech Sc Lomé (Togo).

[7] (1992). Recensement Général de la Population et de l’Habitat (RGPH) 1984. Analyse des résultats définitifs.. Lomé.

[8] Bonita R, De Courten M, Jamrozick K (2001). Surveillance of risk factors for non-communicable diseases: the WHO STEP-wise approach..

[9] Megnigbeto AC, Niakara A, Nebie LVA (2002). Validation of a method of blood pressure measurement for a study of hypertension in a black African population.. Cahiers d’études recherches francophones/santé.

[10] Chobanian VA, Bakris LG, Black HR, Cushman WC, Green LA, Izzo JL (2003). The seventh report of the Joint National Committee on Prevention, Detection, Evaluation, and Treatment of High Blood Pressure.. J Am Med Assoc.

[11] (2009). Cunci 1997??. Echelle brève d’évaluation du stress. Evaluation rapide du stress.. http://www.champsy.org/index.htm..

[12] Tazi MA, Abir-Khalil S, Chouaki N (2003). Prevalence of the main cardiovascular risk factors in Morocco: result of a national survey, 2000.. J Hypertens.

[13] Kane AM, Basa A, Diop AK (1997). Etude clinique des facteurs de risque vasculaire chez l’adulte en milieu rural à Thiadiaye.. Dakar médical.

[14] Ibrahim MM (1995). Hypertension prevalence, awareness treatment, and control in Egypt. Results from the Egyptian National Hypertension Program (BHP). NHP investigate team.. Hypertension.

[15] Mufunda J, Mebrahtu G, Usman A (2005). The prevalence of hypertension and its relationship with obesity: results from a national blood pressure survey in Eritrea.. J Human Hypertens.

[16] Malu K, Baka-Tubia M, Mongolo M (1990). Quelques caractéristiques de l’hypertension, artérielle dans la région Sud-Est du Zaïre.. Cardiologie Tropicale.

[17] Koffi NM, Sally SJ, Kouame P, Silue K, Diarra Nama AJ (2001). Faciès de l’hypertension artérielle en milieu professionnel au port autonome d’Abidjan. Etude prospective à propos de 220 cas.. Méd d’Afrique Noire.

[18] Bertrand E, Akinkugbe OO, Frances Y (1995). Hypertension artérielle des populations originaires d’Afrique Noire. Editions Pradel, Paris?.

